# Rat hepatitis E virus cross-species infection and transmission in pigs

**DOI:** 10.1093/pnasnexus/pgae259

**Published:** 2024-07-01

**Authors:** Kush Kumar Yadav, Patricia A Boley, Carolyn M Lee, Saroj Khatiwada, Kwonil Jung, Thamonpan Laocharoensuk, Jake Hofstetter, Ronna Wood, Juliette Hanson, Scott P Kenney

**Affiliations:** Department of Animal Sciences, Center for Food Animal Health, The Ohio State University, 1680 Madison Ave, Wooster, OH 44691, USA; Department of Veterinary Preventive Medicine, College of Veterinary Medicine, The Ohio State University, 1900 Coffey Road, Columbus, OH 43210, USA; Department of Animal Sciences, Center for Food Animal Health, The Ohio State University, 1680 Madison Ave, Wooster, OH 44691, USA; Department of Animal Sciences, Center for Food Animal Health, The Ohio State University, 1680 Madison Ave, Wooster, OH 44691, USA; Department of Veterinary Preventive Medicine, College of Veterinary Medicine, The Ohio State University, 1900 Coffey Road, Columbus, OH 43210, USA; Department of Animal Sciences, Center for Food Animal Health, The Ohio State University, 1680 Madison Ave, Wooster, OH 44691, USA; Department of Animal Sciences, Center for Food Animal Health, The Ohio State University, 1680 Madison Ave, Wooster, OH 44691, USA; Department of Animal Sciences, Center for Food Animal Health, The Ohio State University, 1680 Madison Ave, Wooster, OH 44691, USA; Department of Veterinary Preventive Medicine, College of Veterinary Medicine, The Ohio State University, 1900 Coffey Road, Columbus, OH 43210, USA; Department of Animal Sciences, Center for Food Animal Health, The Ohio State University, 1680 Madison Ave, Wooster, OH 44691, USA; Department of Animal Sciences, Center for Food Animal Health, The Ohio State University, 1680 Madison Ave, Wooster, OH 44691, USA; Department of Animal Sciences, Center for Food Animal Health, The Ohio State University, 1680 Madison Ave, Wooster, OH 44691, USA; Department of Veterinary Preventive Medicine, College of Veterinary Medicine, The Ohio State University, 1900 Coffey Road, Columbus, OH 43210, USA; Department of Animal Sciences, Center for Food Animal Health, The Ohio State University, 1680 Madison Ave, Wooster, OH 44691, USA; Department of Veterinary Preventive Medicine, College of Veterinary Medicine, The Ohio State University, 1900 Coffey Road, Columbus, OH 43210, USA

**Keywords:** rat, hepatitis E, pigs, infectious, zoonotic

## Abstract

Strains of *Rocahepevirus ratti*, an emerging hepatitis E virus (HEV), have recently been found to be infectious to humans. Rats are a primary reservoir of the virus; thus, it is referred to as “rat HEV”. Rats are often found on swine farms in close contact with pigs. Our goal was to determine whether swine may serve as a transmission host for zoonotic rat HEV by characterizing an infectious cDNA clone of a zoonotic rat HEV, strain LCK-3110, in vitro and in vivo. RNA transcripts of LCK-3110 were constructed and assessed for their replicative capacity in cell culture and in gnotobiotic pigs. Fecal suspension from rat HEV-positive gnotobiotic pigs was inoculated into conventional pigs co-housed with naïve pigs. Our results demonstrated that capped RNA transcripts of LCK-3110 rat HEV replicated in vitro and successfully infected conventional pigs that transmit the virus to co-housed animals. The infectious clone of rat HEV may afford an opportunity to study the genetic mechanisms of rat HEV cross-species infection and tissue tropism.

**Significance Statement**

New zoonotic strains of *Rocahepevirus ratti* [rat hepatitis E virus (HEV)] have emerged infecting both immunocompetent and immunosuppressed people through unknown transmission sources. Pigs are a primary source of transmission for human HEV strains and could serve a similar role for rat HEV transmission as rats are a common pest found on swine farms worldwide. Rats could transmit rat HEV to pigs which could then be transmitted to humans. Determining susceptibility of pigs to emerging zoonotic rat HEV strains can define potentially new transmission routes to inform public health policy and could provide pathology models for rat HEV disease.

## Introduction

Hepatitis E is a disease caused by hepatitis E virus (HEV) (1). HEV is a widespread pathogen found in wild animals (boar, deer) (2, 3), domestic pigs (4, 5), rats (6, 7), chickens (8, 9), and humans (10, 11). The disease in humans is mostly defined as self-limiting but can be lethal in people with underlying health conditions (12, 13), immunosuppressed (14, 15), and during pregnancy (16–19). HEV infection is increasingly being found as a complication for solid organ transplant patients (15, 20–22) where HEV can persist in immunosuppressed individuals, leading to liver cirrhosis (23).

**Fig. 1.**
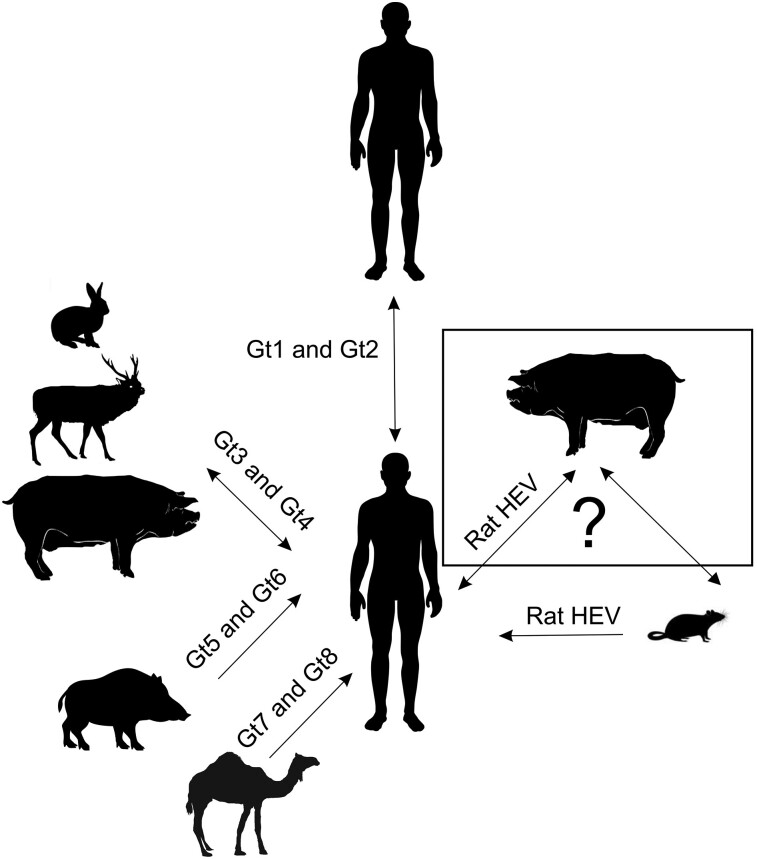
HEV transmission pathways to humans. Paslahepevirus (gt1 and gt2) has been demonstrated to obligately transmit between humans. Paslahepevirus (gt3 to gt8) are considered zoonotic, transmitting from the animal host to humans. New strains of rat HEV have been reported to cause infection in humans via an unknown source of transmission.

HEV is classified in the family Hepeviridae that comprises two subfamilies: Orthohepevirinae and Parahepevirinae (24). Out of four genera (Paslahepevirus, Avihepevirus, Rocahepevirus, and Chirohepevirus) in the Orthohepevirinae, Paslahepevirus is known to cause clinical disease in humans with all eight genotypes potentially capable of infecting humans (gt1 through gt8) (Fig. 1) (19). Gt1 and gt2 are restricted to humans, whereas HEV gt3 through gt8 are zoonotically transmitted from pigs and other species to humans (25). The most common spillover strains are gt3 and gt4 which act as a foodborne illnesses transmitting from primarily pork products to humans (10, 26–29). Multiple studies have demonstrated the presence of HEV in pig slaughter houses (30–32) and in pork products at grocery stores (33–37). Zoonotic transmission of pig gt3 HEV has been a concern for immunosuppressed patients as they develop chronic disease. The clinical outcomes appear to be becoming more severe with more frequent extrahepatic disease manifestations (20, 38, 39).

*Rocahepevirus ratti* (rat HEV), formerly Orthohepevirus C, circulates at a high level in rat populations (40–46). Initial experiments in 2013 with rat HEV concluded it to be noninfectious to humans as evidenced by the absence of viral replication in nonhuman primates (NHPs) (47). Infections in humans were not noted until 2018 when it was first reported in an immunosuppressed human case in Hong Kong (48). Since this initial case, rat HEV has been shown to be infectious to both immunosuppressed (48–51) and immunocompetent humans (52). Rat HEV disease has been defined by mild hepatitis but has been demonstrated to progress to persistent infection in immunosuppressed patients (49). Rat HEV is an international concern as it has been detected in a traveler from Africa to Canada, suggesting a global prevalence of this emerging understudied virus (52). Experimental cross-species transmission studies of rat HEV (V-105) to rhesus and cynomolgus monkeys demonstrated fecal shedding and seroconversion with no significant alteration in liver enzymes (53). Rat HEV recovered from cynomolgus and rhesus monkeys successfully infected both nude and Sprague–Dawley rats (53). Such findings of the cross-species transmission of rat HEV necessitate understanding of the rat HEV host range, transmission to agriculturally important species, and pathogenesis in detail.

There are several areas of concern with emerging rat HEV cases in humans. Serological presence of IgG antibodies against Paslahepevirus did not provide protection against rat HEV (48, 51). Commonly used RT-qPCR screening assays are based on Paslahepevirus; thus, it has been unable to differentially detect rat HEV (48). Retrospective studies to find the transmission source of rat HEV could not identify the exact transmission route of rat HEV to humans (48). Pigs are known as one of the main reservoirs (54) and transmission sources of HEV to humans (55). This emphasizes the need to understand the relevance of this agriculturally important species as a potential intermediate source of rat HEV transmission to humans.

Genetic analyses revealed that all human cases of rat HEV infection reported in Hong Kong are genetically close to LCK-3110 strain (51). In this study, we successfully developed a rat HEV infectious clone (LCK-3110) and demonstrated its ability to develop infection in pigs.

## Results

### Construction of full-length cDNA clone of rat HEV LCK-3110 strain and determination of its replicative ability in cell lines from multiple Species

**Fig. 2.**
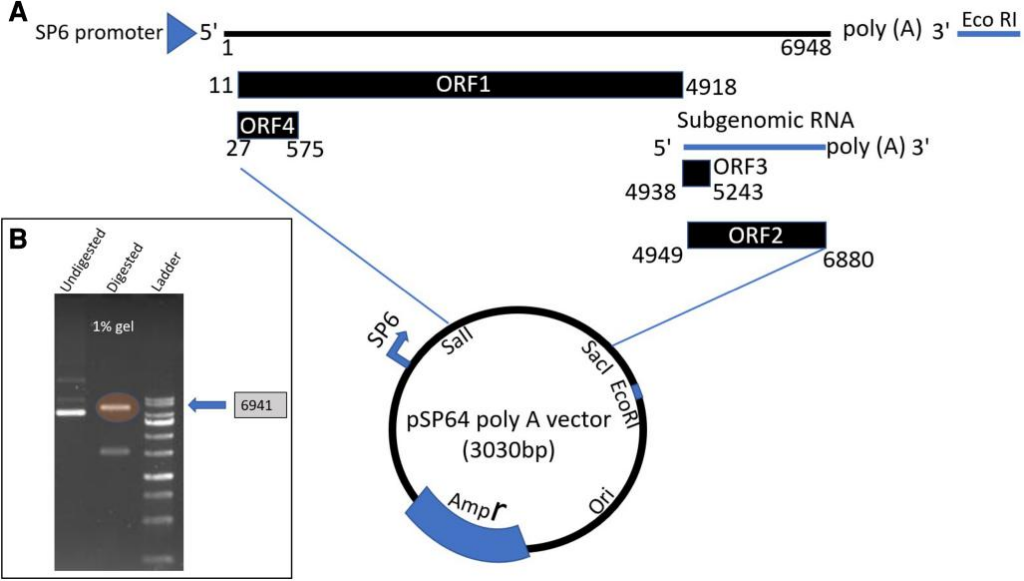
Successful insertion of rat HEV into pSP64 poly (A) vector. A) Schematic representation of full-length genomic rat HEV insertion in the pSP64 poly (A) vector. A cDNA clone of rat HEV LCK-3110 strain was constructed and cloned into the pSP64 poly (A) vector using unique restriction sites, SaII and SacI. It contains EcoRI restriction sites at the 3′ end of the viral genome. B) Results from 1% gel electrophoresis demonstrating the insertion of the full-length rat HEV.

A full-length genomic cDNA clone of rat HEV LCK-3110 strain was constructed and cloned into the pSP64 poly (A) vector (Fig. 2A). The full-length cDNA clone pSP64-rat HEV-poly (A) was sequenced, restriction enzyme digested, and run through agarose gel electrophoresis to check the rat HEV insertion, 6,941 base pairs (Fig. 2B).

**Fig. 3.**
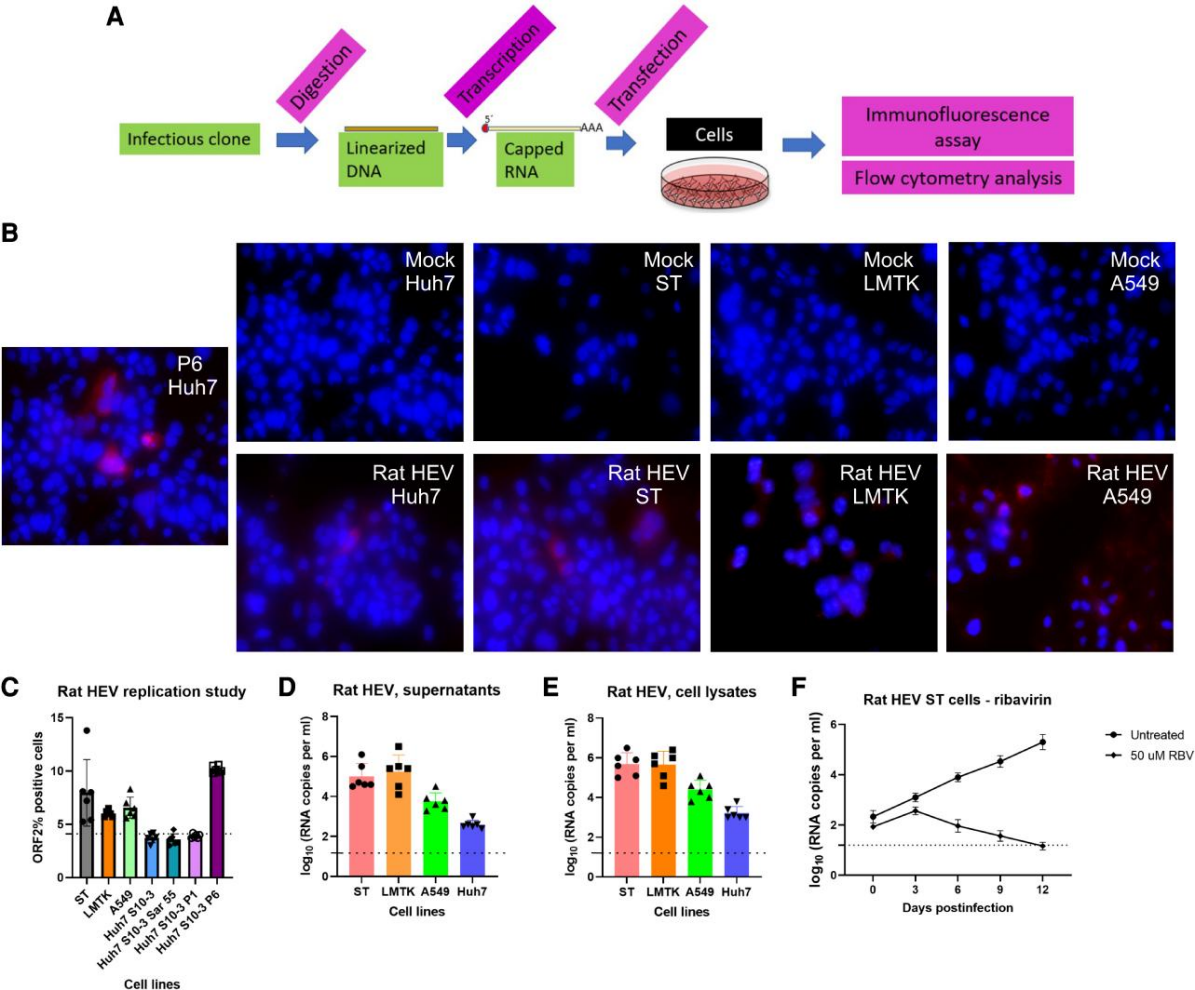
Rat HEV is replication competent in cells. A) Workflow of HEV capping and transfection of target cells. LMTK, A549, Huh7 S10-3, and ST cell lines were transfected with in vitro transcribed capped HEV RNA (rat HEV). Huh7 S10-3 cells were transfected with Kernow-C1 genotype 3 P6 strain and are used as a positive control. B) Immunofluorescence detection of HEV ORF2 antigen in methanol-fixed LMTK, A549, Huh7 S10-3, and ST cells after 5 dpt. Cells are stained with goat antirabbit IgG H&L combined with antirabbit Alexa Fluor 594 (red) and 4′, 6-DAPI (blue). C) Flow cytometry quantification of LMTK, A549, Huh7 S10-3, and ST cells transfected with capped RNA transcripts of rat HEV; Huh7 S10-3 transfected with cell culture-adaptive Kernow-C1 genotype 3 P6 strain and noncell culture-adaptive genotype 1 Sar55 and Kernow-C1 genotype 3 P1 strain. Sar55, P6, and P1 belong to Paslahepevirus which is used as the control to determine the replication ability of rat HEV. The assay was performed in the cells harvested on day 5 post-transfection. Samples were fixed in methanol and probed with rabbit anti-ORF2 followed by goat antirabbit Alexa Fluor 594 antibodies. Each bar (mean ± SD) represents separate transfections stained in parallel and displays the mean of two independent biological experiments with three replicates per sample. D) RT-qPCR data from the supernatants collected from day 5 of replication assay. E) RT-qPCR data from the cell lysates collected from day 5 of replication assay. F) ST cells were transfected with rat HEV LCK-3110 and left untreated or incubated with 50 µM ribavirin (RBV). Samples were collected every 3 days, and viral RNA was measured.

To test the infectivity of the pSP64-rat HEV-poly (A) cDNA clone in vitro, capped viral RNAs were transcribed in vitro (Fig. 3A). The capped RNAs were transfected into human hepatoma (Huh7) subclone 10-3 (S10-3), mouse subcutaneous tissue (LMTK), human carcinoma lung tissue (A549), and swine testicular (ST) cells to determine its replication ability. Cells were fixed and probed for ORF2 expression via an immunofluorescence assay (IFA). As ORF2 is translated from subgenomic mRNA, it is synthesized only during the later stages of HEV replication, serving as an indicator of complete viral replication (56, 57). To demonstrate whether HEV transcripts have productively replicated in the target cells, we assessed events at the single-cell level using IFA and flow cytometry. Employing antibodies (rabbit anti-ORF2 polyclonal serum) directed against Paslahepevirus ORF2 capsid protein, we detected cells expressing the ORF2 protein suggesting replication (Fig. 3B). Total numbers of ORF2 expressing cells were quantified by IFA against ORF2 coupled with flow cytometry. As depicted in Fig. 3C, ST, A549, and LMTK cells were more permissive for rat HEV replication than Huh7 S10-3 cells. Approximately 8, 6.5, 7, and 4% ORF2-positive cells were observed in ST, LMTK, A549, and Huh7 S10-3 cells, respectively. Additionally, Huh7 S10-3 cells were transfected with the cell culture-adapted Kernow-C1 P6 gt3 HEV strain and two noncell culture-adapted strains (Kernow-C1 P1; gt3 and Sar55; gt1) to build a cutoff point to demonstrate the ORF2 percent positive cells. Cell supernatant and cell lysates harvested on day 5 demonstrated viral RNA loads in both cell lysate and supernatants from LMTK, A549, ST, and Huh7 S10-3 cells via RT-qPCR (Fig. 3D, E). To study the replication kinetics overtime, ST cells were transfected with rat HEV LCK-3110 and viral RNA released over time was measured in the cell culture supernatants as described previously in human liver cells (58) and human placental cells (59) in comparison with ribavirin -treated cells. An increase in the viral RNA concentration over time from 2 log_10_ RNA copies/ml to 5 log_10_ RNA copies/mL indicated successful replication (Fig. 3F). Treatment with 50 µM ribavirin successfully inhibited the replication as demonstrated by reduction in the viral RNA copies over time from 2 log_10_ RNA copies/mL to 1.2 log_10_ RNA copies/mL (Fig. 3F). These results demonstrate that the pSP64-rat HEV-poly (A) cDNA clone can produce RNA which is replication competent in vitro.

### Capped RNA transcripts of the rat HEV LCK-3110 were infectious when intrahepatically injected into the livers of gnotobiotic pigs

**Fig. 4.**
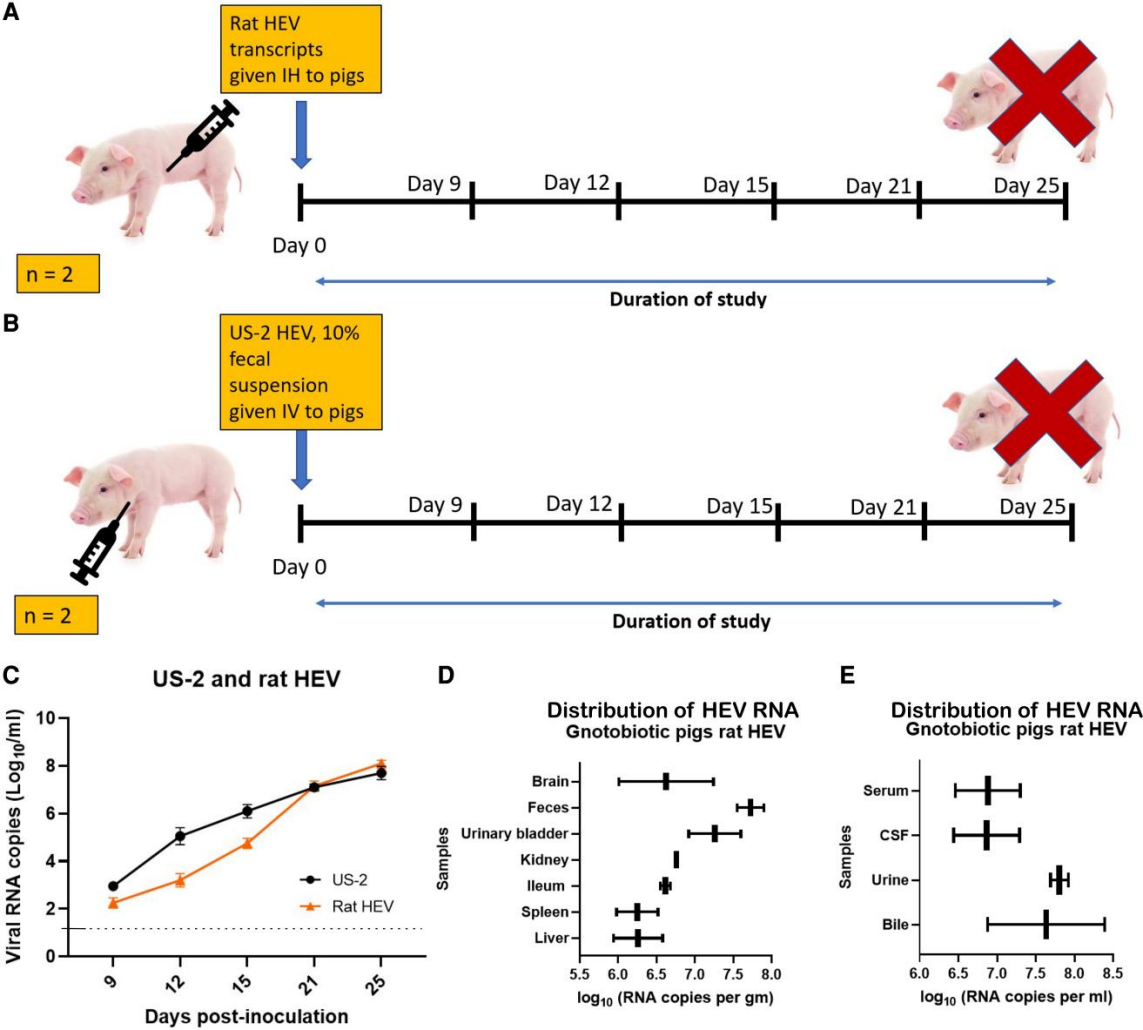
Successful amplification of rat HEV and US-2 HEV in pigs. A) Schematic representation of the experimental design. Capped rat HEV transcripts inoculated intrahepatically (IH) to pigs. B) Schematic representation of the experimental design. Fecal suspension (10%) harvested from US-2-positive pigs inoculated IV to pigs. C) Fecal viral shedding of rat HEV and US-2 HEV. D), E) HEV RNA loads in CSF, brain, feces, urine, urinary bladder, kidney, ileum, spleen, bile, and liver in rat HEV-inoculated pigs.

Since high-titer HEV infectious virus is difficult to obtain in vitro, infectivity or pathogenicity studies with live infectious virus are limited and sometimes result in less interpretable data due to the low starting titer of available infectious virus (60). With the successful construction of an infectious cDNA clone of the rat HEV LCK-3110 strain, we bypassed cell culture virus propagation and amplified the virus directly in animals via an intrahepatic inoculation procedure (61) with capped RNA transcripts. The above-described intrahepatic inoculation procedure (also referred to as in vivo transfection) has been successfully used for pathogenicity studies of swine and human HEV (61–64). Gnotobiotic pigs were intrahepatically inoculated with the LCK-3110-capped viral RNA (1 × 10^5^ viral RNA copies/mL) and tested for evidence of rat HEV infection in the pigs (Fig. 4A). Rat HEV was detected in fecal material starting at day 9 post-inoculation (Fig. 4C). Viral RNA in feces and serum was detected until the study termination date, 35 days post-inoculation (dpi). Rat HEV RNA was also detected in bile, liver, spleen, ileum, kidney, urinary bladder, urine, feces, brain, and cerebrospinal fluid (CSF) (Fig. 4D,E) at 35 dpi. As a positive control, gnotobiotic pigs were inoculated (1 × 10^5^) intravenously (IV) with a 10% fecal suspension from US-2 HEV-infected pigs (kindly provided by Dr. X.J. Meng, Virginia Tech) (Fig. 4B). Evidence of US-2 HEV infection, including fecal virus shedding, was also detected (Fig. 4C).

### Characterization of the pathogenicity of rat HEV LCK-3110 virus in conventional pigs

**Fig. 5.**
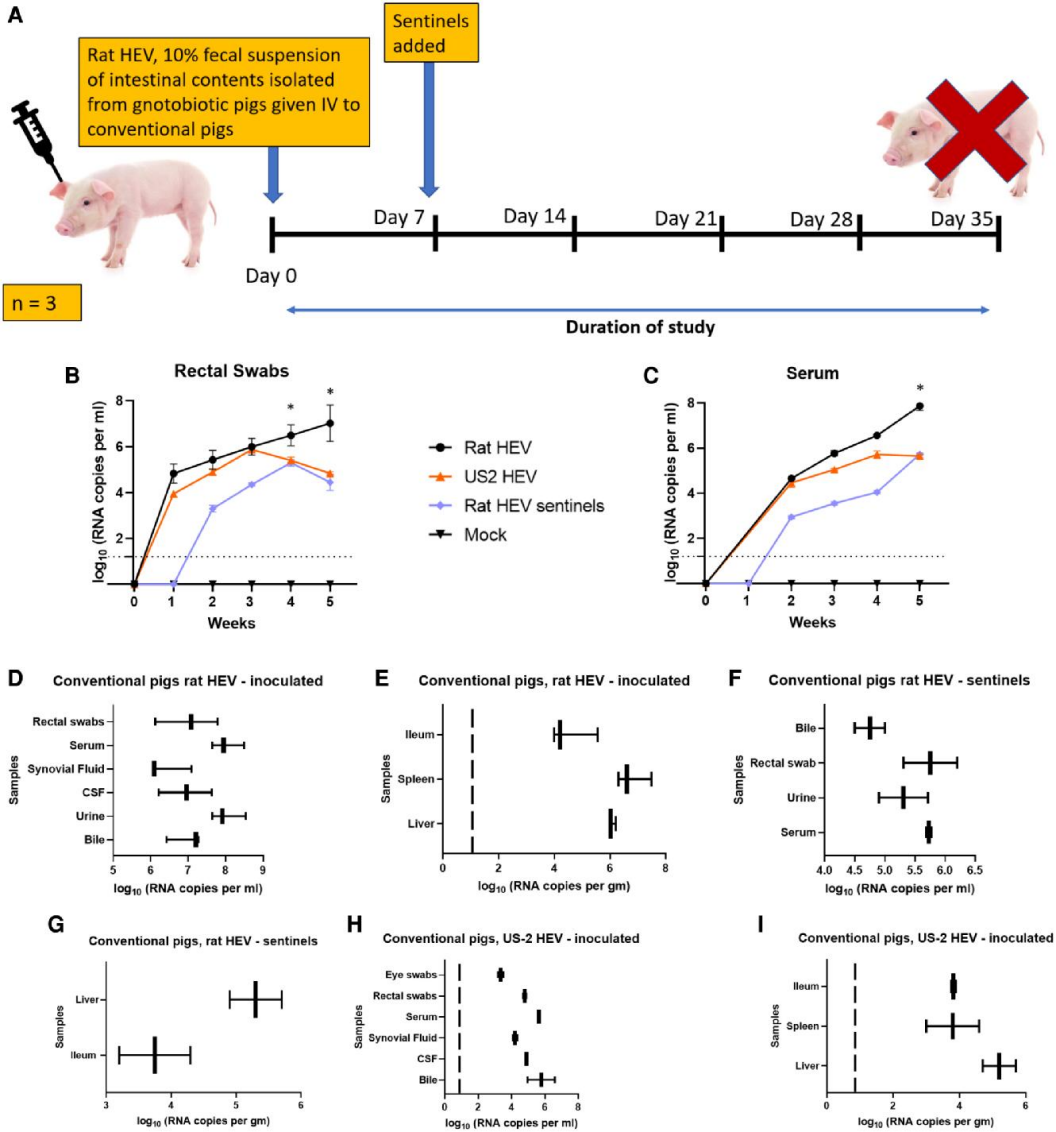
Assessment of the conventional pig model for genotype 1 rat HEV via intravenous inoculation of 10% fecal suspension harvested from gnotobiotic pigs. A) Schematic representation of the experimental design. 10% fecal suspension from rat HEV-inoculated gnotobiotic pigs was used as inoculum for the pathogenesis study. B) Viral RNA in rectal swabs was determined by RT-qPCR between groups. C) Viral RNA in serum was determined by RT-qPCR between groups. D), E) HEV RNA loads in rectal swabs, serum, synovial fluid, CSF, urine, bile, ileum, spleen, and liver in rat HEV-inoculated pigs. F), G) HEV RNA loads in bile, rectal swab, urine, serum, liver, and ileum in sentinel pigs comingled with rat HEV-infected pigs. H), I) HEV RNA loads in eye swabs, rectal swabs, serum, synovial fluid, CSF, bile, ileum, spleen, and liver in US-2 HEV-inoculated pigs.

Intestinal contents derived from the intrahepatically inoculated gnotobiotic pigs sacrificed on day 35 were used for the preparation of an infectious stock used in the pathogenicity study. Group A (*n* = 3) was injected IV with rat HEV (2 × 10^8^ genomic equivalents) (Fig 5A), group B (*n* = 2) was injected IV with US-2 HEV (2 × 10^8^ genomic equivalents), and group C (*n* = 2) was injected IV with phosphate-buffered saline (PBS). Rat HEV LCK-3110 RNA and US-2 HEV RNA were detected in feces (Fig. 5B) and sera (Fig. 5C) by RT-qPCR in group A and group B with primers specific for the LCK-3110 strain and US-2 HEV strain, respectively. Fecal virus shedding and viremia were detected from 1 week post-inoculation in both experimental groups (A and B) with variable rates of detection and rat HEV-inoculated pigs shedding more than the US-2 HEV (Fig. 5). Rat HEV produced a higher fecal titer in pigs at week 1 post-infection than US-2. US-2 fecal shedding appeared to taper off beginning at 3 weeks post-infection, whereas the rat HEV-infected pigs continued to increase fecal shedding through the end of the study, 35 dpi. Sentinel pigs in the rat HEV-infected room began to shed virus in their feces at week 2 post-comingling and appeared to begin tapering off at week 4 post-comingling (Fig. 5B).

Trends in viremia mimicked fecal shedding with rat HEV RNA in the serum increasing for the duration of the study, US-2 RNA peaked at around week 4 post-infection, and rat HEV sentinel animal RNA titers increased for the duration of the study (Fig. 5C). In the rat HEV-infected group, all pigs necropsied on 35 dpi had viral RNA in feces and serum demonstrating fecal viral shedding and viremia. Other samples, from necropsy such as liver, spleen, and ileum were also positive for viral RNA. Bodily fluids such as bile, urine, CSF, and synovial fluid were positive for rat HEV RNA with bile, urine, and feces having the highest concentration of RNA (Fig. 5D, E). Pigs included as sentinels also demonstrated fecal viral shedding and viremia (Fig. 5F). Rat HEV RNA was also seen in bile, urine, and ileum (Fig. 5G).

In the US-2 HEV-infected group, all pigs necropsied at 35 dpi had viral RNA in feces and serum (Fig. 5H). Other samples, such as liver, spleen, ileum, bile, CSF, synovial fluid, and eye swabs, were positive for US-2 HEV (Fig. 5H,I). Mock-challenged animals remained negative for fecal shedding and viremia.

**Fig. 6.**
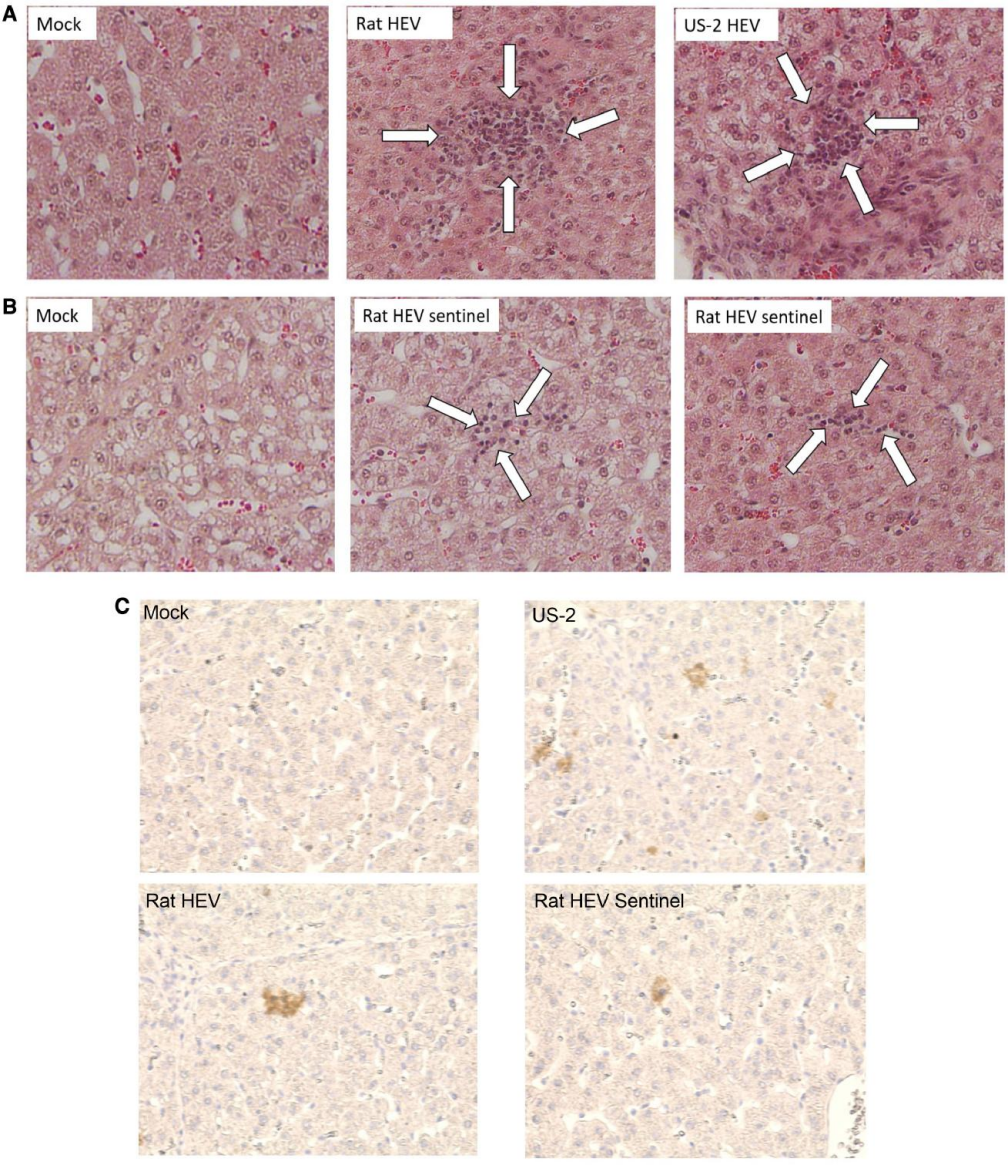
Histopathology and IHC in the liver of pigs inoculated with rat HEV or US-2 HEV and the sentinel pigs at 35 days post-inoculation (dpi). A) H&E-stained liver of a rat HEV-inoculated pig, showing moderate lymphohistiocytic hepatitis (arrows). Note that US-2 HEV-inoculated and mock pigs exhibit moderate lymphohistiocytic hepatitis (arrows) and no lesions, respectively. B) H&E-stained liver of sentinel rat HEV group pigs, showing mild lymphocytic or lymphohistiocytic hepatitis (arrows). Note that the mock pig shows no lesions. C) IHC-stained liver of a rat HEV-inoculated pig, showing a small amount of HEV ORF2 protein (brown stain). Note that US-2 HEV-inoculated and mock pigs exhibit a small amount of HEV ORF2 protein (brown stain) and no IHC-positive cells, respectively. The rat HEV sentinel pig also has a small amount of HEV ORF2 protein in the liver. Chromogenic detection of HEV ORF2 protein via 3,3′-diaminobenzidine (DAB) staining. Original magnification (A–C), all × 400. H&E- or IHC-stained images were taken by Keyence BZ-X800.

In HEV-inoculated pigs, histopathological lesions were limited to the liver. Both rat HEV- and US-2 HEV-inoculated pigs had moderate, multifocal lymphohistiocytic hepatitis at 35 dpi (Fig 6A). Compared with rat HEV-inoculated pigs, the sentinel pigs had less infiltration of nonsuppurative inflammatory cells, demonstrating milder hepatitis or early status of inflammatory responses to HEV infection (Fig. 6B). By immunohistochemistry (IHC), HEV ORF2 protein was identified in the livers of rat HEV and US-2 HEV-inoculated pigs. The HEV ORF2 protein was seen mainly in the cytoplasm or perinuclear region of hepatocytes at 35 dpi (Fig. 6C). Mock-infected pigs occasionally had mild splenic or hepatic congestion, but they did not exhibit HEV ORF2 protein in the tissues tested.

**Fig. 7.**
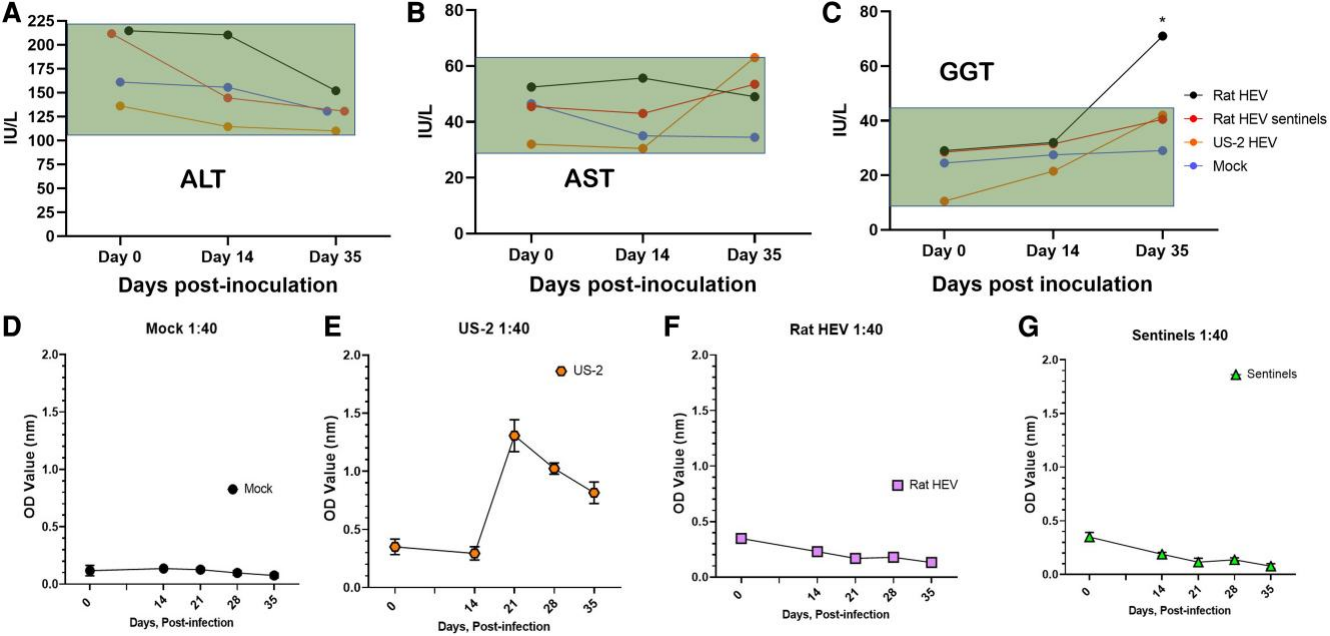
Liver function enzyme levels and seroconversion to HEV during rat HEV infection. The serum biochemistry of ALT (A), AST (B), and GGT (C) was examined in rat HEV-infected pigs (*n* = 3), rat HEV sentinels (*n* = 2), US-2 HEV-infected (*n* = 2), and mock pigs (*n* = 2). The values inside the green block represent the normal expected range of the enzymes in pigs. An increase in the GGT by day 35 is seen in the pigs inoculated with the rat HEV. All values are shown in units per liter (IU/L). D) Negative control pigs did not show any seroconversion by day 35. E) In positive control pigs (US-2 HEV inoculated), the titer of the anti-HEV antibody increased after day 14. F, G) No seroconversion was seen in rat HEV-inoculated pigs and rat HEV sentinel pigs.

The liver enzyme tests indicated no significant differences between the groups in alanine aminotransferase (ALT) and aspartate aminotransferase (AST) during the period of infection, suggesting that no serious liver damage was induced in the pigs by rat HEV infection (Fig. 7A,B). The levels of gamma-glutamyl transferase (GGT) were higher in rat HEV-inoculated pigs by day 35, indicating the initiation of cholestasis in pigs (Fig. 7C). Total antibodies against HEV were examined in all groups from days 0, 14, 21, 28, and 35. Negative control pigs did not seroconvert throughout the study (Fig. 7D). Interestingly, even though US-2 HEV-inoculated pigs seroconverted by day 21 (Fig. 7E), pigs inoculated with rat HEV did not seroconvert (Fig. 7F). Rat HEV sentinels did not seroconvert (Fig. 7G) similar to rat HEV-inoculated pigs.

### Rat HEV transmission through fecal–oral route to sentinel pigs

The fecal–oral route is considered as one of the major routes of transmission of HEV. To study whether the virus is efficiently transmitted to sentinel pigs, naïve animals were co-housed with pigs that had been infected with the fecal inoculum. Sentinel pigs added to the rat HEV-inoculated group on 7 dpi started shedding virus in feces after 7 days post-contact (Fig. 5B). Viremia (Fig. 5C) was evident in the sentinel pigs as early as 7 days post-contact. High titers of viral RNA were detected in the feces and blood of these animals, although these titers were lower than those of the originally infected pigs. However, only bile, urine, liver, and ileum demonstrated viral RNA after 28 days post-contact with the rat HEV-inoculated pigs (Fig. 5F, G). Overall, these data confirm the efficient transmission of rat HEV between co-housed animals.

### Human hepatoma cell lines Huh7 S10-3 and HepG2/C3A support LCK-3110 replication

**Fig. 8.**
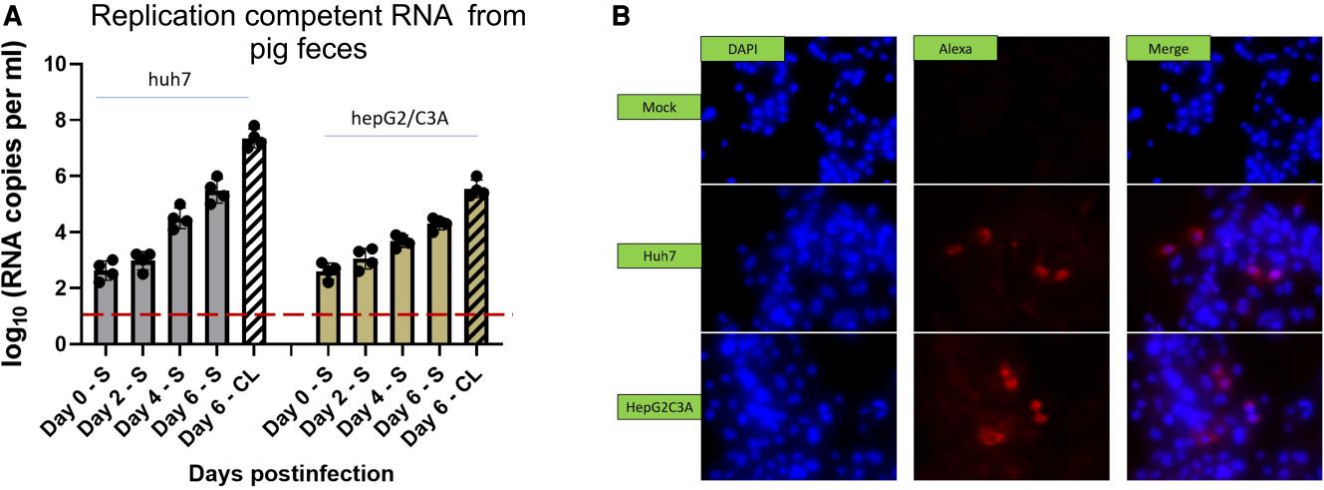
Isolation of rat HEV from infected pig feces in cell culture. A) Rat HEV RNA loads in culture supernatant (S) and cell lysates (CL) of Huh7 S10-3 and HepG2/C3A cell lines after inoculation by filtered fecal suspension from rat HEV-inoculated pigs. Independent biological experiments, mean ± SD of 4 replicates, are presented. Red line represents the cutoff value demonstrating the background referring to the attachments of the virus to the cell surfaces. B) Immunofluorescence detection of HEV ORF2 antigen in methanol-fixed Huh7 S10-3 and HepG2/C3A cells after 6 dpi. Cells were stained with rabbit anti-ORF2 and goat antirabbit IgG H&L combined with antirabbit Alexa Fluor 594 (red) and 4′, 6-DAPI (blue).

Huh7 S10-3 and HepG2/C3A were inoculated with LCK-3110 rat HEV obtained from intestinal contents of rat HEV-inoculated conventional pigs. We detected rat HEV RNA in supernatants from both cell lines (Fig. 8A) inoculated with pig intestinal contents on days 2, 4, and 6. RNA detected on day 0 (6 h post-inoculation) was considered as background with attachment of the virus to the cell surfaces. RNA loads in cell lysates collected on day 6 demonstrated successful viral cell entry and replication (Fig. 8A). IFA (Fig. 8B) of Huh7 S10-3 and HepG2/C3A cells on day 6 post-inoculation confirmed the presence of ORF2 protein in the inoculated cells. Replication in Huh7 S10-3 cells was comparatively better than HepG2/C3A cells (Fig. 8A). These results demonstrate the ability of rat HEV derived from conventional pig feces to replicate in human hepatoma cell lines.

### Rat liver cells (clone 9) are susceptible to rat HEV LCK-3110 strain

**Fig. 9.**
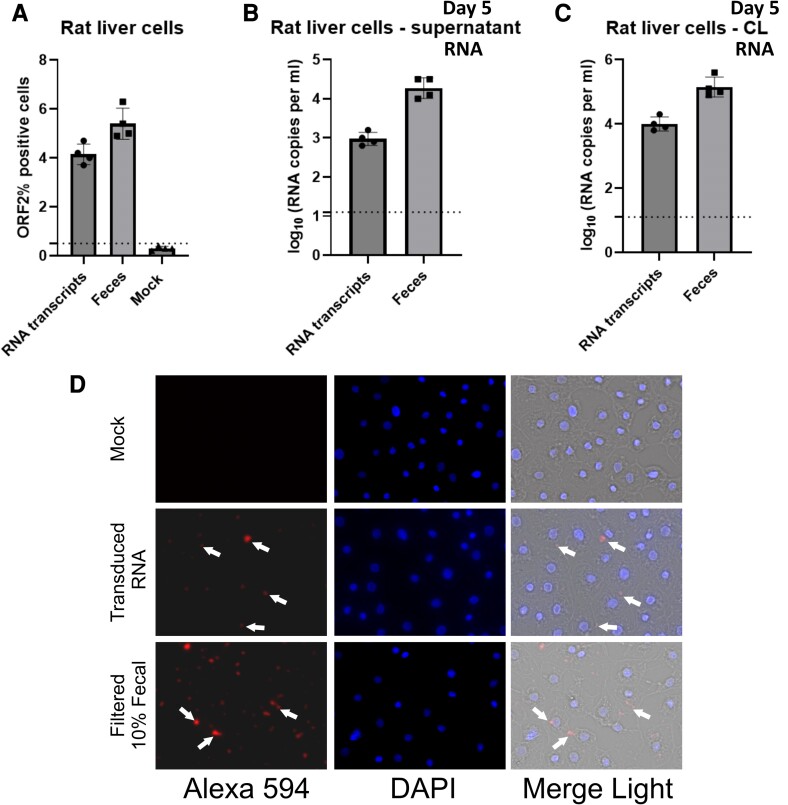
Rat liver cells (clone 9) susceptibility to LCK-3110 strain. A) Flow cytometry quantification of rat liver (clone 9) cells transfected with capped RNA transcripts of rat HEV. The assay was performed in the cells harvested on day 5 post-transfection. Samples were fixed in methanol and probed with rabbit anti-ORF2 followed by goat antirabbit Alexa Fluor 594 antibodies. Each bar (mean ± SD) represents separate transfections stained in parallel and displays the mean of two independent biological experiments with four replicates per sample. Rat HEV RNA loads in B) culture supernatant (S) and C) cell lysates (CL) of rat liver cell lines after rat HEV RNA transfection and inoculation of filtered fecal suspension from rat HEV-inoculated pigs. Independent biological experiments, mean ± SD of 4 replicates, are presented. Dotted line represents the cutoff value demonstrating the background referring to the attachments of the virus to the cell surface. D) Immunofluorescence detection of HEV ORF2 antigen in methanol-fixed rat liver cells after 5 dpt. Cells are stained with goat antirabbit IgG H&L combined with antirabbit Alexa Fluor 594 (red) and 4′, 6-DAPI (blue).

Rat liver cells (clone 9) were transfected with rat HEV transcripts and inoculated with LCK-3110 rat HEV obtained from the intestinal contents of rat HEV-inoculated conventional pigs. We observed ORF2-positive cells using IFA and flow cytometry. As demonstrated in Fig. 9A, approximately 3.8% of the cells were ORF2 positive when transfected with LCK-3110 RNA transcripts (1 × 10^5^ viral RNA copies/ml) and 4.5% of the cells were ORF2 positive when inoculated with 10% fecal suspension from rat HEV-inoculated conventional pigs (∼1 × 10^7^ viral RNA copies). Cell supernatant and cell lysates harvested on day 5 demonstrated viral RNA loads in supernatants from rat liver cells via RT-qPCR (Fig. 9B, C). We also detected cells expressing ORF2 protein (Fig. 9D).

## Discussion

Increasing HEV host range is a topic of considerable concern (65). Historically, all HEV infections in humans have been attributed to Paslahepevirus (gt1 to gt8), but recently *R. ratti* has been identified as a causative agent in human disease (48–51). In 2013, rat HEV strain LA-B350 isolated from rats in US (GenBank: KM516906.1) was considered noninfectious to humans based on the genetic diversity from Paslahepevirus strains and its inability to infect NHPs and pigs (47, 66). However, from 2017 to 2022, 13 hepatitis E cases attributed to rat HEV have been identified, and to date, most of them were in Hong Kong. Genetic analyses indicated that these strains were close to LCK-3110 strain (GenBank: MG813927.1) (51). The LCK-3110 strain has significantly diverged from LA-B350 with 200/1,633 amino acid changes in the ORF1 polyprotein, 50/644 amino acid changes in the ORF2 protein, and 36/102 amino acid changes in ORF3 proteins (data not shown). Further experimental genetic studies are required to understand how LCK-3110 has overcome the previous human species susceptibility block. In our study, we developed the LCK-3110 infectious clone and explored its infectious ability to known primary reservoirs of Paslahepevirus HEV, namely pigs. Our results reveal that pigs can serve as an intermediate host for rat HEV infection in humans and should be studied further for this transmission potential.

Other recent studies determining rat HEV cross-species transmission potential showed the V-105 strain of *R. ratti* isolated from Vietnam was capable of infecting primates (53). Strain V-105 is genetically closest to the LCK-3110 strain group and shares 93.7% nucleotide identity with LCK-3110. In our study, we demonstrate that the LCK-3110 strain can infect pigs producing suppurative hepatitis and shedding virus with no significant alterations in liver enzymes as seen in cynomolgus and rhesus monkeys with V-105 (53). In addition, previously studied strains of rat HEV (R4, R8, and LA-B350) lacked replication in rat liver cell lines such as N1-S1, clone 9, MH1C1, and HAIIE (67, 68). Interestingly, we demonstrated that LCK-3110 has some ability to replicate in clone 9 rat liver cells. This could be because the rat HEV strain was isolated from an immunosuppressed transplant patient in comparison with other studies where it was isolated directly from rats (67, 68). Further studies are necessary to understand the underlying genetic mechanisms leading to enhanced cross-species replication abilities. These findings demonstrate that rat HEV LCK-3110 may transmit from humans to rats demonstrating a potential for reverse zoonoses.

*R. ratti* strains have been detected in rats from Japan (6), United States (47), Germany (67, 69), Indonesia (46), Vietnam (70), and many other countries, suggesting a geographically widespread distribution of rat HEV among rat populations. Although nonpersistent infection has been described in rats with rat HEV (42), higher rates of progression to persistent rat HEV infection have been reported in immunosuppressed humans even after reductions in immunosuppressive drug therapy (50). This finding is concerning and needs to be further studied to understand how to best treat rat HEV infection in immunosuppressed humans utilizing in vivo models.

Multiple studies describing rat HEV in humans have reported no contact of the patients with rats (48, 49). Retrospective studies to identify the transmission source of rat HEV to humans failed to demonstrate the presence of rat HEV in the organs of local rats, from swab samples of drains, or in rat fecal samples. Only the internal organs from one rat harvested in 2012 were reported positive for rat HEV (48). This emphasizes that there could be an intermediate source for rat HEV infection that is mediating the transmission of the virus to humans.

Some studies have investigated commensal rats and pigs in proximity to the residence of rat HEV-positive humans (49), which might have contributed to infection. Only 7/159 *Rattus norvegicus* reported positive for rat HEV (49). All 172 pig rectal swabs were negative for rat HEV RNA (49). The findings do not definitively eliminate pigs as a transmission source due to the low level and transient nature of HEV RNA shed and inability to serologically separate rat HEV from Paslahepevirus HEV strains. Pigs are well-known reservoirs of Paslahepevirus HEV strains without producing major clinical signs and could complicate serosurveillance studies. Time of sample collection and viral load in the commensal rats are limitations during RNA surveillance studies. Another study demonstrated two *Rattus rattus* that were positive for both rat HEV and genotype 3 swine HEV. Swine HEV detected in rats was identical to the swine HEV in pigs. The detection of HEV in the liver is an indication of active viral replication, as the liver is the major organ of viral replication (71). Rats and pigs can easily be exposed to each other's feces containing high amounts of virus. Thus, the probability of pigs being infected with rat HEV via contaminated feces cannot be eliminated. With our experimental finding that pigs can be infected with rat HEV, it further enhances our knowledge of potential rat HEV host range, necessitating further study on this possible transmission vector. A recent epidemiological study demonstrated the presence of rat HEV in pigs at different farm locations in Cordoba, southern Spain enhancing the feasibility of a role of pigs in the transmission of rat HEV to humans (72). More surveillance and the development of rat HEV specific serological tools are needed to appropriately address pigs as potential transmission vectors for rat HEV.

Our study demonstrates the presence of viral RNA in the CSF of rat HEV-infected pigs. The recent discovery of HEV invasion of microvascular endothelial cells and the ability to cross the blood–brain barrier invading the nervous system demands the need to study rat HEV effects in the central nervous system (73). Meningoencephalitis followed by death has been reported in an immunosuppressed organ transplant recipient with persistent rat HEV infection. Examination of the CSF revealed the presence of rat HEV (49).

One aberration in our infection data was a lack of detectable seroconversion in LCK-3110-infected pigs or sentinels. Investigations of rat HEV antigenicity show that the highly divergent rat HEV sequence when compared with Paslahepevirus reduces the ability of defined diagnostic assays to detect infection. Failure of cross protective antibodies against rat HEV even when previously exposed to Paslahepevirus necessitates an update to the existing diagnostic assays for HEV (51). Our ELISA utilized amino acids 391–620 which encompassed the region 455–603 of ORF2 known as the protruding domain which is the immunodominant epitope in HEV (74). The average intergenotypic amino acid identity within Paslahepevirus strains is 89.5%, while the amino acid identity with LCK-3110 is only 48% (51). Within our epitope region, this trend remains constant with a 47.4% amino acid identity to LCK-3110. The reduced sensitivity of the Paslahepevirus ORF2 bait protein could be a reason for the lack of seroconversion seen in our rat HEV-inoculated pigs. However, differences in seroconversion rates have been demonstrated in pigs with various HEV strains. Pigs experimentally infected with swine HEV gt3 seroconverted in 55 days, whereas pigs infected with US-2 HEV seroconverted within a week (75). We found similar results for US-2 HEV, but there was no seroconversion seen in the rat HEV group despite viral shedding in feces, viremia, and presence of HEV in the liver. We speculate that the time allocated for the study was possibly not sufficient to produce significant seroconversion in the rat HEV-inoculated pigs.

We did not see major elevations of liver enzymes such as ALT and AST in the US-2-infected pigs validating previous findings (75). ALT and AST levels in the rat HEV-inoculated pigs were no different than US-2 HEV and mock pigs. However, GGT levels were significantly higher in the rat HEV-inoculated group. Increased GGT activity is an indicator of cholestasis in pigs (76) and is used in the investigation of hepato-pancreatic or renal disorders (77). Rat HEV-positive asymptomatic human patients were described with very mild liver dysfunction matching our results in the animal model (50).

## Conclusion

In conclusion, we have constructed an infectious cDNA clone of a genotype 1 LCK-3110 strain of rat HEV and demonstrated its infectivity in vitro and in vivo. We produced infectious viruses through intrahepatic RNA inoculation of gnotobiotic pigs and characterized the pathogenicity of rat HEV through intravenous injection of intestinal contents to conventional pigs. Like Paslahepevirus HEV infection in pigs, gross lesions were not detected with only microscopic lesions evident in the liver of rat HEV-infected pigs. Furthermore, we revealed that pigs could serve as an intermediate source of emerging zoonotic rat HEV transmission to humans.

## Materials and methods

### Construction of full-length cDNA clone of rat HEV

The full-length genomic sequence of the LCK-3110 strain of rat HEV (GenBank MG813927.1) was artificially synthesized (Genscript). The plasmid pSP64 poly (A) vector (Promega) was used to clone the full-length rat HEV genome between the SalI and SacI sites (Fig. 2A). After the successful insertion, the plasmid was transformed into stable *E. Coli* (New England Biolabs, NEB) and grown overnight at 37 °C in the presence of ampicillin.

### Linearization of plasmid DNA

For linearization, plasmid DNA encoding rat HEV was linearized using EcoRI (NEB). Five percent of the reaction was subjected to gel electrophoresis with ethidium bromide staining and visualized with ultraviolet light to verify that linearization had occurred.

### In vitro transcription for IFA

Viral capped mRNA (rat HEV) was made from linearized DNA using the Promega Ribomax Large Scale RNA Production System SP6 (Promega PRP 1300) and ARCA CAP (TriLink Biotechnologies). The fidelity of transcripts was assessed and normalized by agarose gel electrophoresis.

### Cell culture

LMTK (isolated from mouse subcutaneous tissue, ATCC:CCL-1.3), A549 (isolated from human lung carcinoma, ATCC:CRM-CCL-185), ST (isolated from ST, ATCC:CRL-1746), Huh7 (human hepatoma cells) S10-3 subclone (78, 79) (kindly provided by Dr. X.J. Meng), and rat liver cells clone 9 (isolated from liver of rat, ATCC:CRL-1439) and HepG2/C3A (derivative of HepG2, ATCC HB-8065) were used for the study. LMTK, Huh7 S10-3, HepG2/C3A cells, and ST cells were cultured in Dulbecco modified Eagle's medium (DMEM) containing 10% fetal bovine serum (FBS). A549 cells and clone 9 were cultured in F12/K media containing 10% FBS.

### Ribavirin

Ribavirin was purchased from ChemDirect. It was stored at 4°C and diluted according to the instructions by the manufacturer. It was used as an HEV RNA replication inhibitor (59).

### Transfection of ST, LMTK, Huh7 S10-3, and A549 cells

Cells were seeded to acquire a seeding density of 2 × 10^6^ cells. 16 μL of 1 × 10^5^ viral RNA copies/ml was utilized for RNA transfection using a Mirus Trans-IT mRNA transfection kit. After 48 h of transfection, cells were passaged 1:3 to three new wells and incubated for an additional 3 days.

### Flow cytometry of in vitro transcribed capped *R. ratti* HEV RNA-transfected (ST, Huh7 S10-3, LMTK, and A549) cells

Five days post-transfection (dpt), cells were trypsinized and pelleted. Cells were then fixed in 200 μL of 100% methanol at 4 °C and stored at −80 °C. Cells were centrifuged out of methanol, washed, and resuspended in PBS. Cells were blocked-in blocking solution [5% nonfat dried milk, 0.1% Triton X-100 in PBS (PBST)] in a 96-well plate for 30 min at 37 °C. Cells were then washed with PBS once before probing with primary antibody—rabbit antitruncated open reading frame (ORF)2 HEV (57, 66) diluted 1:50 in blocking solution for 30 min at 37 °C. ORF2 encodes for structural capsid protein of HEV. We synthesized the truncated ORF2 recombinant protein using *E. coli* and generated in-house primary antibody against the ORF2 protein in rabbits. Amino acids 391–620 which encompassed the regions 455–603 of ORF2 known as the protruding domain which is the immunodominant epitope in HEV (74) were used in the study. After washing twice with PBS, cells were incubated with secondary antibody—goat antirabbit phycoerythrin (PE) (Life Technologies) diluted to 1:200 in PBS for 30 min at 37 °C. Cells were then washed twice with PBS and resuspended in 200 µL of PBS. Fluorescence was analyzed for 100,000 events using a flow cytometer (BD Accuri C6 Plus, Biosciences, San Diego, CA, USA). Gates were set to exclude dead cells, doublet discrimination based on forward and side scatter profiles, and mock-infected cells were used to gate background fluorescence (79).

### Indirect immunofluorescence

At 5 dpt, transfected cells were fixed in 100% cold methanol, permeabilized with PBST, and blocked with 5% nonfat milk (Sigma-Aldrich, St. Louis, MO, USA). Immunostaining of ORF2-encoded capsid protein was performed using a 1:200 blocking buffer-diluted rabbit antitruncated ORF2 HEV antibody for 30 min at 37°C. Cells were washed three times with PBST. A fluorescently labeled goat antirabbit IgG H&L antibody (Alexa Fluor 594; abcam, Cambridge, FL, USA) was used at a dilution of 1:400 in PBS to detect bound primary antibodies. 4′, 6-Diamidino-2-phenylindole (DAPI) was used to stain the nucleus. For quantification of virus infectivity, wells were manually observed with a fluorescent microscope (Keyence) for specific fluorescence, and the presence of fluorescent foci was recorded. A fluorescent focus was defined as a minimum of one to two cells showing clear intracytoplasmic fluorescence (79).

### Intrahepatic inoculation of capped RNA transcripts from rat HEV infectious cDNA clone

All animal experiments in this study were approved by the Ohio State University Institutional Animal Care and Use Committee (IACUC 2020A00000068), and virus studies were approved by the Ohio State Institutional Biosafety Committee (IBC 2016R00000082). Four gnotobiotic pigs from 1 sow were derived near term and maintained in two different sterile isolation units (two per unit) as described previously (80). On day 2, pigs were administered capped rat HEV transcripts via intrahepatic injection. The other two pigs were IV inoculated with 10% fecal suspension from US-2-positive pigs (positive control). Rectal swabs from pigs were collected on days 9, 12, 15, 21, and 25. Pigs were humanely euthanized on day 25. Blood, intestinal contents, tissues (brain, urinary bladder, kidney, duodenum, jejunum, ileum, pancreas, liver, bile, spleen), and bodily fluids (CSF, urine, bile) were harvested from rat HEV-inoculated pigs.

### Intravenous inoculation of 10% fecal suspension of rat HEV in conventional pigs

Nine conventional pigs negative for rat HEV and US-2 HEV RNA in rectal swabs were randomly divided into three groups (A, B, C). Group A (*n* = 3) was IV administered 10% fecal suspension derived from rat HEV-inoculated gnotobiotic pigs. Group B (*n* = 2) was IV administered 10% fecal suspension derived from US-2 HEV-inoculated gnotobiotic pigs. Group C (*n* = 2) was IV administered PBS. After a week, two pigs negative for rat HEV were added as sentinels with group A (rat HEV). Rectal swabbing was done weekly in all groups. Blood collection was done on days 0, 14, 21, 28, and 35. Pigs were humanely euthanized on day 35. Blood, intestinal contents, eye swabs, tissues (brain, urinary bladder, kidney, duodenum, jejunum, ileum, pancreas, liver, bile, spleen, lungs, skeletal muscle), and bodily fluids (CSF, urine, bile) were harvested from the pigs.

### RNA extraction and RT-qPCR

RNA extraction was performed using Trizol reagent (Invitrogen) from harvested cell supernatant and cell lysates on day 5 from A549, LMTK, Huh7 S10-3, and ST cells. Similarly, RNA was extracted from serum, blood, eye swabs, homogenized tissues, and bodily fluids. Reverse transcriptase quantitative polymerase chain reaction (RT-qPCR) was performed. A one-step RT-qPCR was carried out using TaqMan Fast Virus 1-step Master Mix (Thermo Scientific) under a protocol of 50 °C for 15 min, 95 °C for 2 min, and 45 cycles of 95 °C for 5 seconds and 60 °C for 30 seconds (Mastercycler RealPlex). A forward primer rat HEV F, 5′-CTTGTTGAGCTYTTCTCCCCT-3′, a reverse primer, 5′-CTGTACCGGATGCGACCAA-3′, and a probe 5′-FAM-TGCAGCTTGTCTTTGARCCC-Dabcyl-3′ were used for the detection of rat HEV. A 10-fold serial dilution of the capped rat HEV RNA (10^7^ to 10^1^ copies) was used as the standard for the quantification of the viral genome copy numbers.

A similar procedure was done for pig samples experimentally infected with US-2 HEV. A forward primer US-2 HEV F, 5′-GGTGGTTTCTGGGGTGAC-3′, a reverse primer, 5′-AGGGGTTGGTTGGATGAA-3′, and a probe 5′-FAM-TGATTCTCAGCCCTTCGC-Dabcyl-3′ were used for the detection of US-2 HEV. A 10-fold serial dilution of the capped US-2 HEV RNA (10^7^ to 10^1^ copies) was used as a standard for the quantification of the viral genome copy numbers.

### Histological examinations and IHC

The liver (from each lobe), pancreas, duodenum, jejunum, ileum, and spleen of virus- or mock-infected pigs were collected and fixed in 10% neutral-buffered formalin. Tissues were embedded, sectioned (3.5 µm), and stained with Gill's hematoxylin and eosin (H&E) for light microscopic examination as described previously (81). Formalin-fixed tissue sections were tested by IHC for the detection of HEV, as previously described with slight modifications (82). The rabbit anti-HEV ORF2 antibody was used as the primary antibody, and a horseradish peroxidase-conjugated antirabbit antibody (BioGeneX) was used for visualization as brown staining. Stained tissues were counterstained with hematoxylin.

### Recombinant proteins

Two nucleotide sequences encoding for amino acids 391–620 of gt3 ORF2 (74) were codon optimized for bacterial expression and synthesized commercially (Integrated DNA Technologies, IDT), inserted into bacterial T7 expression vector pRSETa (Invitrogen), and expressed. Recombinant protein was produced using BL21 (DE3) chemically competent cells via autoinduction. Proteins were analyzed via SDS–PAGE and western blot. The bacteria were lysed with B-Per reagent (ThermoFisher) (5 mL/g) with 1 mM ethylenediaminetetraacetic acid. ELISA protein was solubilized and purified with Ni-NTA columns followed by dialysis. Protein was quantified using Bradford assay.

### ELISA

ELISAs were modified and optimized to detect HEV ORF2-specific IgG antibodies in serum. Five µg/mL of purified plasmid lysate diluted in carbonate buffer (20 mM Na_2_CO_3_, 20 mM NaHCO_3_, pH 9.6) was bound to Nunc Maxisorp 96-well plates (ThermoFisher) at 50 µL per well at 4 °C overnight. The following morning, after washing, 150 µL of blocking buffer (4% nonfat dried milk in PBS with 0.1% Tween [PBST 0.1%]) was added to the antigen-coated wells and incubated for 2 h at 37 °C. Plates were washed with PBS, and 50 µL of serum was heat inactivated at 56 °C for 30 minutes. Inactivated serum was 2-fold serially diluted in blocking buffer and added to each well. The plates were incubated for 1 h at 37 °C. After washing, 50 µL of HRP-conjugated secondary antibody (antiswine [Sigma]) in 4% non-fat dry milk (NFDM)/PBST (0.1%) at a dilution of 1:200 K was added and incubated at 37 °C for 1 h. Wells were washed with PBST (0.1%) five times between each step. 3,3′,5,5′-Tetramethylbenzidine substrate (Seracare) was added and incubated for approximately 10 minutes, and the reaction was stopped by adding 50 µL of 0.3 mol/L sulfuric acid. Plates were read at an absorbance of 450 nm using a SpectraMax F5 plate reader (Molecular Devices). All experiments were done under the same conditions with each sample tested three times.

### Liver enzyme levels

ALT, AST, and GGT values in the pig's sera were monitored using VETSCAN VS2 (Zoetis). The geometric mean titer of ALT, AST, and GGT in negative control animals was considered the normal ALT titer, and a fold greater or more was considered as alteration in the liver enzymes.

### Virus culture

Huh7 S10-3 and HepG2/C3A cells were seeded in six-well plates at 2 × 10^5^ cells per well in 2 mL of DMEM with 10% FBS and penicillin (100 units/mL) and streptomycin (100 g/mL) and incubated at 37 °C for 24 h. The infections were performed utilizing a 10% fecal suspension diluted 1:5 in DMEM and 0.45 μm filtered. Culture media were removed, and cells were inoculated with 1 mL of the resulting solution (1 × 10^7^ viral RNA copies). At room temperature, plates were rocked for 1 h and then incubated at 37 °C for 6 h. The inoculum was removed, and fresh culture media were added. Supernatants were collected on days 0, 2, 4, and 6. Supernatants and lysates were tested for rat HEV via RT-qPCR.

### Statistical analyses and reproducibility

All quantitative data are presented with the mean and SD. Analyses of independent data were performed by Student's unpaired two-tailed *t* test. Statistical analyses were carried out using GraphPad Prism 9.4.1. *P* < 0.05 was considered significant.

## Data Availability

All experimental data described in this study are included in the main text.

## Preprints

This manuscript was posted on a preprint [bioRxiv 2023.07.06.547957; doi: https://doi.org/10.1101/2023.07.06.547957].
